# Percutaneous mitral balloon commissurotomy in juvenile rheumatic mitral stenosis patients: a ten-year experience from Ethiopia

**DOI:** 10.1186/s12872-026-05770-4

**Published:** 2026-03-25

**Authors:** Mohammed Bedru Sebah, Azene Dessie Mengistu, Tilahun Dessie Alene, Abdu Endris Assefa, Adisu Meles Kabtyimer

**Affiliations:** 1 Cardiac Center Ethiopia, Addis Ababa, Ethiopia; 2 Cardiac center Ethiopia and Saint Paul’s hospital millennium medical college (SPHMMC), Addis Ababa, Ethiopia; 3 Department of pediatrics and child health, School of medicine, College of Medicine and Health Science, Wollo University, Dessie, Ethiopia; 4 Department of Epidemiology and Biostatistics, School of Public Health, College of Medicine and Health Science, Wollo University, Dessie, Ethiopia

**Keywords:** Percutaneous mitral balloon commissurotomy, Juvenile patients, Rheumatic mitral stenosis, Ethiopia

## Abstract

**Background:**

Rheumatic heart disease (RHD) remains a major cause of acquired valvular heart disease in low- and middle-income countries, disproportionately affecting young people. The mitral valve is most frequently involved, and severe rheumatic mitral stenosis (MS) represents the dominant presentation. MS is a progressive disease that gradually impairs cardiac output and, if untreated, leads to pulmonary hypertension, right-sided heart failure, and premature death. Percutaneous mitral balloon commissurotomy (PMBC) is considered the first-line intervention for symptomatic severe MS with favorable valve anatomy. This study aimed to assess the clinical, echocardiographic, and procedural outcomes of PMBC among juvenile patients with rheumatic MS in Ethiopia.

**Methods:**

A Descriptive cross-sectional study was conducted at the Cardiac Center Ethiopia, Addis Ababa, from July to August 2025. All consecutive patients who underwent PMBC during the study period and fulfilled eligibility criteria were included (*n* = 86). Data were analyzed using STATA 17. Continuous variables were expressed as means ± SD or medians (IQR), and categorical variables as frequencies and percentages. Pre- and post-procedure echocardiographic and clinical parameters were compared using paired statistical tests. Associations between baseline characteristics and immediate outcomes were examined using Fisher’s exact tests.

**Results:**

The median age was 15 years (± 5), and 60.5% (*n* = 52) were female. Patients originated from multiple Ethiopian regions, predominantly Amhara 28 (32.6%) and Oromia 25 (29.1%), with 65.06% residing in Rural areas. Severe MS accounted for 84 (97.7%) of cases, while 2 patients (2.3%) presented with severe restenosis. 82 (95.4%) had a mitral valve score ≤ 8. The mitral valve area increased significantly from 0.6 cm² (IQR: 0.26) to 1.5 cm² (IQR: 0.6), and the mean mitral gradient decreased from 31.4 ± 8.3 mmHg to 16.4 ± 5.6 mmHg (both *p* < 0.001). Procedural success was achieved in 85 cases (98.84%).

**Conclusion:**

PMBC in juvenile patients with severe rheumatic MS in Ethiopia is a safe and effective intervention, demonstrating significant improvements in mitral valve area and mean mitral gradient, with a high procedural success rate.

## Introduction

Rheumatic heart disease (RHD) remains a leading cause of acquired valvular heart disease in low- and middle-income countries, with an estimated global burden of 50 million cases and over 360,000 deaths annually [1], disproportionately affecting young populations in endemic regions. The heaviest burden is seen in sub-Saharan Africa, South Asia, and parts of the Pacific, where access to preventive care and cardiac surgery is limited [1, 2].

In Ethiopia, RHD is the predominant cause of valvular heart disease and a major driver of morbidity and premature mortality among adolescents and young adults, particularly women of reproductive age [3, 4]. The mitral valve is the most commonly affected in rheumatic heart disease, with severe rheumatic mitral stenosis (MS) being the predominant manifestation [5]. MS is a progressive condition that gradually impairs cardiac output and, if left untreated, ultimately leads to heart failure and death unless mechanical intervention is performed to enlarge the mitral valve orifice and reduce left atrial pressure [6].

Percutaneous mitral balloon commissurotomy (PMBC) also termed percutaneous mitral commissurotomy or balloon mitral valvuloplasty is a catheter-based technique first developed by Inoue in 1984, which splits fused commissures and enlarges the mitral orifice [7]. PMBC is the first-line intervention for symptomatic severe rheumatic MS with favorable valve morphology, in the absence of left atrial thrombus or severe mitral regurgitation (MR) [8]. Compared with surgical commissurotomy or valve replacement, PMBC is less invasive, avoids prosthesis-related complications such as lifelong anticoagulation, and is repeatable—advantages that are particularly critical in resource-limited settings. Regional evidence from East Africa supports the effectiveness and safety of PMBC. In a recent prospective cohort study from Tanzania, interventional therapy including percutaneous balloon mitral valvuloplasty and surgery was associated with significantly lower mortality compared with medical management in patients with rheumatic mitral stenosis over a median follow-up of 23.5 months [9–12].

Large international registries report procedural success rates of over 90% with low periprocedural mortality. Documented complications include acute severe mitral regurgitation, cardiac tamponade, stroke, vascular complications, and late restenosis. Long-term follow-up studies show sustained clinical improvement in the majority of patients, although atrial fibrillation and a high Wilkins score predict worse outcomes.

In Ethiopia, however, PMBC is available only at the country’s single dedicated children cardiac referral center in Addis Ababa, making access very limited, Moreover, local evidence regarding procedural outcomes and their clinical and echocardiographic determinants remains scarce, highlighting an important gap in context-specific data [13].

Given the ongoing high burden of RHD in Ethiopia, combined with limited surgical resources, additional well-documented chart reviews on immediate clinical, echocardiographic, and procedural outcomes of PMBC can fill an important evidence gap. Even without long-term follow-up, such analyses provide benchmarks for success, guide local patient selection, and inform peri-procedural planning in resource-limited settings. So, the aim of this study was to evaluate the clinical, echocardiographic, and procedural outcomes before and after percutaneous mitral balloon commissurotomy (PMBC) in juvenile patients with rheumatic mitral in Ethiopia.

## Methods

### Study setting and design

Ethiopia, with an estimated population of over 120 million, carries a significant burden of rheumatic heart disease (RHD), yet access to advanced cardiac care remains extremely limited, specialized cardiac treatment centers in the country are scarce, with the Children’s Heart Fund of Ethiopia Cardiac Center in Addis Ababa being the only fully dedicated cardiac referral and treatment facility. This makes it a national hub, receiving patients from all regional states.

The Children’s Heart Fund of Ethiopia (CHFE) in Addis Ababa, is the only specialized cardiac referral facility in the country. The center delivers comprehensive, around-the-clock cardiac care for both pediatric and adult patients through a multidisciplinary team comprising cardiologists, cardiac surgeons, anesthesiologists, intensivists, and specialized nursing staff. With a capacity of 30 inpatient beds, a dedicated intensive care unit, and a fully equipped cardiac catheterization laboratory, the center provides both diagnostic and interventional services. Since its establishment, CHFE has emerged as the cornerstone of cardiovascular care in Ethiopia, performing life-saving open-heart surgeries, catheter-based procedures such as percutaneous mitral balloon commissurotomy (PMBC), and offering long-term follow-up and rehabilitation services for patients with rheumatic and congenital heart diseases.

### Study design

A Descriptive cross-sectional study design was employed at the CHFE.

### Source population

The source population comprised all patients diagnosed with severe mitral stenosis who were evaluated for PMBC at the CHFE.

### Study population

The study population included patients with severe mitral stenosis who underwent PMBC at the CHFE during the study period and met the eligibility criteria.

### Study period

The data were collected between July, 2025 and August, 2025.

### Sampling technique and sample size

A consecutive sampling technique was employed, whereby patients who underwent PMBC during the study period and fulfilled the inclusion criteria were included in the study for symptomatic severe rheumatic mitral stenosis between *2015 and 2024* A total of 86 patients of 6–18 age years old and both sexes met the inclusion criteria and were analyzed.

### Eligibility criteria

#### Inclusion criteria

All juvenile patients with symptomatic severe rheumatic mitral stenosis who underwent percutaneous mitral balloon commissurotomy (PMBC) during the study period were included. Patients were eligible regardless of the presence of associated cardiac conditions, including severe tricuspid regurgitation, moderate mitral regurgitation, moderate aortic valve lesions, and right or left ventricular systolic dysfunction. 

#### Exclusion criteria

Patients with left atrial thrombus and those with more than moderate mitral regurgitation were excluded from the study [14].

### Procedural details

PMBC was performed using the Inoue balloon technique under fluoroscopic and echocardiographic guidance.

### Independent variables

The independent variables considered in this study were grouped into three categories. Sociodemographic and baseline characteristics included region, place of residence, sex, age, weight, and height. mitral valve area (pre- and post-procedure), mitral mean gradient (pre- and post-procedure), tricuspid regurgitation gradient (pre- and post-procedure), tricuspid regurgitation velocity (pre-procedure), however, post-procedure TR velocity was not available in the medical records and therefore could not be analysed, tricuspid regurgitation (TR) grade (pre- and post-procedure), mitral regurgitation grade (pre- and post-procedure), Wilkins mitral valve score, tricuspid annular plane systolic excursion, left atrial size/volume, ejection fraction, aortic regurgitation grade, history of stroke, and atrial fibrillation. Finally, procedural variables included balloon inflation size/diameter and number of inflations.

### Dependent variable

Successful PMBC.

### Operational definition

#### Successful PMBC

Procedural success was defined as achieving a post-procedure mitral valve area (MVA) greater than 1.5 cm² **or** at least a two-fold increase in mitral valve area from baseline, or a reduction in the mean mitral valve gradient by at least 50%, and no increase in mitral regurgitation beyond grade 2, with an increase of no more than one grade among individuals with baseline moderate mitral regurgitation [15].

#### Failed PMBC

Those who didn’t fulfill successful PMBC criteria.

#### Severe rheumatic mitral stenosis

Severe mitral stenosis was defined as a mitral valve area < 1.0 cm² and/or a mean transmittal gradient > 10 mmHg, in the presence of typical rheumatic valve morphology [16].

### Data collection

Data were collected using a structured and pretested questionnaire designed in alignment with the study objectives. Trained physicians and research assistants extracted data from patient charts and catheterization records. The tool captured demographic information (age, sex), clinical data, echocardiographic parameters (mitral valve area, mean mitral gradient, commissural morphology, and degree of mitral regurgitation), and post-procedural outcomes including immediate complications.

To maintain data quality, investigators reviewed completed forms daily for completeness and accuracy. Missing or inconsistent entries were cross-checked against the original patient charts. Data were double-entered and cleaned prior to analysis. Patient identifiers were excluded, and all information was kept strictly confidential and used only for this study.

### Statistical analysis

Data were entered and analyzed using STATA version 17. Continuous variables were assessed for normality using the Shapiro–Wilk test, supplemented by graphical inspection. Variables with approximately normal distributions were summarized using means and standard deviations (SD), whereas non-normally distributed variables were summarized using medians and interquartile ranges (IQR). Categorical variables were expressed as frequencies and percentages.

Comparisons between pre- and post-procedure echocardiographic and clinical parameters were performed using paired t-tests for normally distributed continuous variables. For variables that did not satisfy normality assumptions, the Wilcoxon signed-rank test was applied. Associations between baseline characteristics and procedural success were examined using Fisher’s exact test, given the small number of unsuccessful outcomes.

## Result

### Socio-demographic and baseline characteristics of participants

A total of 86 patients who underwent PMBC were included in the analysis. The median age of participants was 15 ± 5 with range 6–18 years. The majority of patients 44.19% were within the 16–18 years age group, followed by those aged 13–15 years (26.74%). Females accounted for 60.47% (*n* = 52) of the study population.

Regarding place of residence, patients were drawn from multiple regions of Ethiopia, with the largest proportion from Amhara (32.56%), followed by Oromia (29.07%) and 62.79% were from Rural. The mean weight and the median height of participants were 36.12 ± 1.083 kg and 1.515 ± 0.14 m, respectively (Table 1).

**Table 1:** Sociodemographic characteristics of RHD patients with MS who had PMBC at CHFE, Addis Ababa, 2015–2024 (*n* = 86)

	Category	Frequency	Percent
Age of participant	6–9	4	4.65
	10–12	21	24.42
	13–15	23	26.74
	16–18	38	44.19
Region	Addis Ababa	11	12.79
	Afar	1	1.16
	Amhara	28	32.56
	Dire Dawa	1	1.16
	Gambella	2	2.33
	Harar	1	1.16
	Oromia	25	29.07
	SNNP	13	15.12
	Tigray	4	4.65
Sex	Female	52	60.47
	Male	34	39.53
Residence	Rural	54	62.79
	Urban	29	33.72
	Missing (.)	3	3.49

### Clinical and echocardiographic characteristics of participants

Among the 86 patients, 97.67% of patients presented with severe mitral stenosis, and 2 (2.33%) presented with severe mitral restenosis and 2.3% had aortic regurgitation, 20.9% had mild, and 7.0% had moderate regurgitation. History of stroke was present in 3.5% of patients, atrial fibrillation in 1.2%, and spontaneous echo contrast in 7.0%. Normal TAPSE and EF were observed in 95.4% and 96.5% of patients, respectively, while 95.4% had a mitral valve score ≤ 8. In terms of hemodynamic outcomes (Table 2).

**Table 2:** Clinical and Echocardiographic pre- -PMBC Hemodynamic characteristics of RHD patients With MS who had PMBC at CHFE, Addis Ababa, 2015–2024 (*n* = 86)

	Frequency (*n*)	Percentage (%)
Aortic Regurgitation grade		
No	60	69.8
Trace	2	2.3
Mild (1–1.5)	18	20.9
Moderate (2)	6	7.0
History of Stroke		
No	83	96.5
Yes	3	3.5
Tricuspid Annular Plane Systolic Excursion (TAPSE)		
Normal	82	95.4
Reduced	4	4.6
Ejection Fraction (EF)		
Normal	83	96.5
Reduced	3	3.5
Atrial Fibrillation		
Present	1	1.2
Absent	85	98.8
Spontaneous Echo Contrast (SEC)		
No	80	93.0
Yes	6	7.0
Mitral Valve Score (Wilkins)		
Favorable (≤ 8)	82	95.4
Grey zone (9–11)	1	1.2
Unfavorable (≥ 12)	3	3.5

The median mitral valve area increased from 0.6 cm² (IQR: 0.26) before the procedure to 1.5 cm² (IQR: 0.6) after the procedure, (z = 8.056, *p* < 0.001). Similarly, the mean mitral gradient decreased from 31.4 ± 8.3 mmHg pre-procedure to 16.4 ± 5.6 mmHg post-procedure (mean difference − 15.1 mmHg, 95% CI: − 16.5 to − 13.7, *p* < 0.001). The median mitral valve mean pressure was also reduced, from 20 mmHg (IQR: 9) to 6 mmHg (IQR: 3) (z = − 8.057, *p* < 0.001). In addition, the tricuspid regurgitation gradient declined from a median of 66.5 mmHg (IQR: 45) to 40 mmHg (IQR: 22) (z = − 7.848, *p* < 0.001). The mean pre-procedure tricuspid regurgitation velocity was 4.1 ± 1.1 m/s (Table 3).

**Table 3:** Clinical and Echocardiographic pre- and post-PMBC Hemodynamic characteristics of RHD patients With MS who had PMBC at CHFE, Addis Ababa, 2015–2024 (*n* = 86)

Parameter	Pre-PMBC (Median [IQR] or Mean ± SD)	Post-PMBC (Median [IQR] or Mean ± SD)	Test Statistic	*p*-value
Mitral Valve Area (cm²)	0.6 [0.26]	1.5 [0.6]	z = 8.056	< 0.001
Mean left atrial pressure (mmHg)	31.4 ± 8.3	16.4 ± 5.6	t = − 21.44	< 0.001
Mitral Valve Mean Gradient (mmHg)	20 [9]	6 [3]	z = − 8.057	< 0.001
TR Gradient (mmHg)	66.5 [45]	40 [22]	z = − 7.848	< 0.001
TR Velocity (m/s)	4.1 ± 1.1	—	—	—

**Fig. 1:**
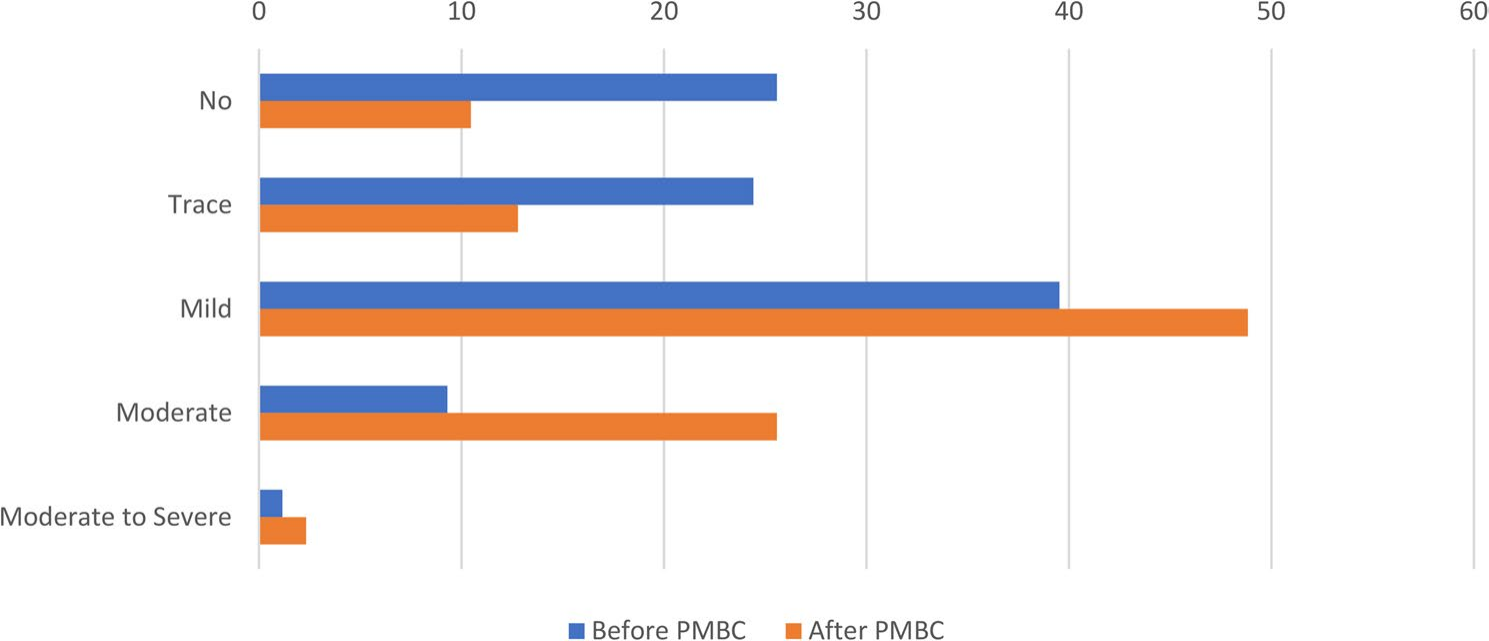
Mitral Regurgitation Grading Pre and Post PMBC RHD patients With MS who had PMBC at CHFE, Addis Ababa, 2015–2024 (*n* = 86)

The distribution of MR grades before and after PMBC. Prior to the procedure, the majority of patients had mild MR, followed by no MR and trace MR. After PMBC, there was a noticeable shift in MR severity. The proportion of patients with mild MR increased, and the number of patients with moderate MR also rose compared to baseline. Conversely, the proportions of patients with no MR and trace MR decreased following the intervention. Only a small number of patients had moderate-to-severe MR both before and after PMBC (Fig. 1).

### Outcomes of percutaneous mitral balloon commissurotomy

Procedural success was observed in 85 (98.84%) cases, with post-intervention Mitral Valve Area ranging from 0.8 to 2.8 cm^2^ in successful outcomes. Conversely, 1 patient (1.16%) experienced unsuccessful procedures.

### Fisher’s exact test results for pre- and post-PMBC hemodynamic characteristics

No statistically significant associations were identified between baseline demographic, clinical, or echocardiographic characteristics and procedural success. Fisher’s exact test was applied instead of the chi-square test due to the highly imbalanced outcome distribution, with only one failure event, which violated the assumptions required for chi-square analysis (Table 4).

**Table 4:** Fishers Exact test result for pre- and post-PMBC Hemodynamic characteristics of RHD patients With MS who had PMBC at CHFE, Addis Ababa, 2015–2024 (*n* = 86)

Variable	Category	Not Success (*n*)	Success (*n*)	Fisher’s Exact *p*-value
Region of Residence	Addis Ababa	0	11	0.198
	Afar	0	1	
	Amhara	0	23	
	Oromia	1	22	
	SNNP	0	9	
	Tigray	0	4	
AR grading categories	No	1	59	1
	Trace	0	2	
	Mild	0	18	
	Moderate	0	6	
MR grading baseline	No	0	22	1
	Trace	0	21	
	Mild	1	33	
	Moderate	0	8	
	Moderate+	0	1	
MR grading post procedure	No	0	9	0.512
	Trace	0	11	
	Mild	0	42	
	Moderate	1	21	
	Moderate+	0	2	
MV score (Wilkins score)	Favorable	1	81	1
	Grey zone	0	1	
	Unfavorable	0	3	
Atrial fibrillation	AF	0	1	1
	No AF	1	84	
Baseline diagnosis	Severe MR	0	2	1
	Severe MS	1	83	
Sex	Female	1	51	1
	Male	0	34	
Spontaneous eco contrast	No	1	79	1
	Yes	0	6	
Ejection Fraction	Normal	1	82	1
	Reduced	0	3	

## Discussion

Rheumatic heart disease (RHD) continues to impose a major burden in low- and middle-income countries, where children and adolescents are disproportionately affected [17]. In Ethiopia, many patients present late with advanced mitral stenosis, often during adolescence, due to inadequate early detection and limited access to secondary prophylaxis [18]. The present study provides new evidence on the immediate outcomes of percutaneous mitral balloon commissurotomy (PMBC) in a juvenile cohort, a population for which data remain scarce in sub-Saharan Africa.

In this study, the majority of patients 52 (60.47%) were female, which is consistent with findings from other studies in Ethiopia (78%), reflecting the high burden of rheumatic mitral stenosis among females [19]. Most participants were from rural areas 54 (65.06%), and the largest proportion were residents of the Amhara region 28 (32.56%), underscoring the continued predominance of rheumatic heart disease in rural populations where access to early diagnosis and treatment remains limited [20]. The incidence of atrial fibrillation (AF) was 1 (1.2%), which is substantially lower than rates reported in other Ethiopian studies (20.3%) and lower than those observed in studies from India (15.7%) which may be due to the difference in the study population and sample size [13, 19, 21]. With respect to mitral regurgitation, most patients had none to mild MR at baseline, with an increase in mild to moderate MR following PMBC, a pattern consistent with previous reports and reflecting the expected hemodynamic effect of commissural splitting during the procedure [22, 23].

The overall procedural success rate in this study was 98.84%, which is higher than that reported in previous studies among juvenile patients from East Africa, Tanzania and South Asia that reported success rates 83.3% and 87.7% respectively [24, 25]. also, this rate was slightly higher than those reported in Ethiopian adult cohorts, where success rates exceeded 90% [13, 19]. The higher success in our study may be attributed to several factors. Valve morphology was relatively favorable in the majority of patients, and our study population included many younger patients, whose mitral valve tissue may be more compliant. In addition, increased opportunities for operator training have enhanced procedural capacity in the region, accompanied by growing regional experience in managing technically challenging cases [26, 27].

Hemodynamic improvements were substantial. The mitral valve area increased from a median of 0.6 cm² to 1.5 cm², mean left atrial pressure decreased from 31.4 ± 8.3 mmHg to 16.4 ± 5.6 mmHg, and the mitral valve gradient was significantly reduced. These results confirm the ability of PMBC to rapidly relieve obstruction and restore normal left atrial pressure, consistent with pediatric and adult studies from Ethiopia, Tanzania and Nepal [19, 24, 25, 28]. In addition, the significant reduction in tricuspid regurgitation gradient supports the beneficial unloading effect on the right heart, echoing previous findings that PMBC can reverse pulmonary hypertension in children and adolescents [29]. By separating these observations, the results more clearly highlight the spectrum of hemodynamic benefits across both left- and right-sided chambers.

With respect to safety, no patients developed severe iatrogenic mitral regurgitation requiring urgent surgery. This aligns with reports that younger valves, being more pliable and less calcified, are less prone to Para commissural tearing [30]. The absence of severe complications reinforces the safety profile of PMBC in juveniles. Nonetheless, the durability of these results is uncertain. Because Restenosis can happen [31], which highlights the importance of long-term follow-up and reinforcement of rheumatic fever prophylaxis programs in Ethiopia.

No statistically significant associations were observed between baseline demographic, clinical, or echocardiographic variables and procedural success. Fisher’s exact test was used instead of the chi-square test because the outcome distribution was highly imbalanced, with one failure event, violating chi-square assumptions. The lack of significant associations is likely attributable to the small sample size, the very high procedural success rate, and reliance on bivariable analyses, which together limited statistical power to detect meaningful differences.

Taken together, this study demonstrates that PMBC is highly effective and safe in juvenile patients with severe mitral stenosis.

## Conclusion

Percutaneous mitral balloon commissurotomy is a highly effective and safe intervention for juvenile patients with severe rheumatic mitral stenosis in Ethiopia. In this cohort, PMBC achieved an excellent procedural success rate with marked and immediate hemodynamic improvements, including significant enlargement of mitral valve area, reduction in transmitral gradient, and lowering of left atrial and tricuspid regurgitation gradients. These findings demonstrate meaningful relief of both left-sided obstruction and secondary right-sided pressure overload.

The very low rate of complications, including the absence of severe iatrogenic mitral regurgitation, underscores the safety of PMBC in children and adolescents, particularly in those with favorable valve morphology. The high success observed in this juvenile population likely reflects younger, more compliant valve tissue and growing procedural expertise within the region.

Given the continued burden of rheumatic heart disease in rural and resource-limited settings, PMBC should remain the preferred first-line therapy for symptomatic severe rheumatic mitral stenosis in juveniles. Future studies with larger sample sizes and longitudinal follow-up are needed to evaluate long-term outcomes and durability of PMBC in this population.

### Implications

These findings support wider adoption of PMBC for pediatric and adolescent patients with severe rheumatic MS in Ethiopia and similar low-resource settings. Early intervention may prevent progression to pulmonary hypertension, right heart failure, and premature mortality. Moreover, the high success and safety profile underscore the need to expand access to specialized cardiac centers and training programs to optimize outcomes in young patients with rheumatic heart disease.

### Strength and limitation of the study

This study addresses an important and under-represented clinical area by providing context-specific evidence on percutaneous mitral balloon commissurotomy outcomes in juvenile patients with rheumatic mitral stenosis in Ethiopia. Strengths include the focus on a high-burden, low-resource setting, acceptable single-center cohort size, and the use of standardized echocardiographic parameters to demonstrate clinically meaningful hemodynamic improvements. However, as a descriptive study, it is inherently limited by inability to establish causality, potential selection and information bias, and restricted generalizability beyond similar settings. The single-center design, small number of failure events, and absence of long-term follow-up further limited assessment of predictors of outcome and durability of benefit.

## Data Availability

The datasets used and/or analyzed during the current study are available from the corresponding author on reasonable request.

## Abbreviations

CHFE: Children’s Heart Fund of Ethiopia
IQR: Interquartile range
MVA: Mitral valve area
MR: Mitral regurgitation
MS: Mitral stenosis
PMBC: Percutaneous mitral balloon commissurotomy
RHD: Rheumatic heart disease
SD: Standard deviation
STATA: Statistical software
TR: Tricuspid regurgitation
